# Modulatory Interactions of Resting-State Brain Functional Connectivity

**DOI:** 10.1371/journal.pone.0071163

**Published:** 2013-08-30

**Authors:** Xin Di, Bharat B. Biswal

**Affiliations:** Department of Biomedical Engineering, New Jersey Institute of Technology, University Heights, Newark, New Jersey, United States of America; West China Hospital of Sichuan University, China

## Abstract

The functional brain connectivity studies are generally based on the synchronization of the resting-state functional magnetic resonance imaging (fMRI) signals. Functional connectivity measures usually assume a stable relationship over time; however, accumulating studies have reported time-varying properties of strength and spatial distribution of functional connectivity. The present study explored the modulation of functional connectivity between two regions by a third region using the physiophysiological interaction (PPI) technique. We first identified eight brain networks and two regions of interest (ROIs) representing each of the networks using a spatial independent component analysis. A voxel-wise analysis was conducted to identify regions that showed modulatory interactions (PPI) with the two ROIs of each network. Mostly, positive modulatory interactions were observed within regions involved in the same system. For example, the two regions of the dorsal attention network revealed modulatory interactions with the regions related to attention, while the two regions of the extrastriate network revealed modulatory interactions with the regions in the visual cortex. In contrast, the two regions of the default mode network (DMN) revealed negative modulatory interactions with the regions in the executive network, and vice versa, suggesting that the activities of one network may be associated with smaller within network connectivity of the competing network. These results validate the use of PPI analysis to study modulation of resting-state functional connectivity by a third region. The modulatory effects may provide a better understanding of complex brain functions.

## Introduction

Large-scale functional brain connectivity studies have provided a better understanding of the human brain functions [1], [2]. After the discovery that the motor regions exhibited highly synchronized functional magnetic resonance imaging (fMRI) signals without explicitly performing a motor task [3], large body of studies on functional connectivity are based upon the resting-state fMRI paradigm. The regions that are in the same functional system typically reveal high functional connectivity in the resting-state [4], [5], [6]; thus, different brain systems can be identified based on the functional connectivity and relative independence of different brain regions [7], [8], [9], [10].

The functional connectivity measures are generally based upon the temporal correlation of the resting-state fMRI time series [11], which implicitly assumes a stable relationship over time. However, accumulating studies have shown the time-varying properties of functional connectivity [12], [13], [14], [15], [16] as well as variations in spatial distribution of the brain networks [17], [18]. Many researchers have suggested that these dynamic changes in connectivity should also be systematically explored since such property may provide a better understanding of the brain functions in both resting-state and task dependent conditions [19], [20], [21], [22], [23]. In the present study, we investigated the modulation of functional connectivity between two regions by a third region to examine one possible mechanism of dynamic functional connectivity.

In general, functional connectivity is measured based on correlations between fMRI time series, and can be expressed as a linear regression model:
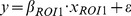
where 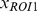 represents the time series of a seed region, 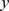 represents the time series of a given voxel, ε represents the residual. The model parameter 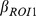 thus represents the relationship (functional connectivity) between the seed region and the given voxel. Friston and colleagues proposed to include two regions of interest (ROIs) and their interaction (also known as physiophysiological interaction, PPI) in the regression model to examine the modulatory interaction effect [24]. Subsequently, the regression model can be expressed as the following:



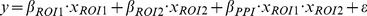
where 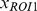 and 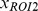 denote the time series of the two ROIs. The critical term of this model is the interaction 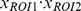. This model can be rewritten as the following:



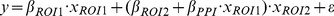



This illustrates that the relationship between the resultant time series 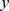 and the ROI2 time series 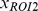 is expressed as 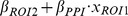, which is a linear function of the ROI1 time series 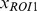. If the interaction effect 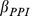 is significant, it will imply that the relationship between the resultant region and the ROI2 depends on the activation of ROI1.

The interaction effect is calculated at the neuronal level by deconvolving the BOLD signals with a hemodynamic response function (HRF) [25]. And the PPI analysis is conducted in a voxel-wise basis to identify regions that showed modulatory interactions with the two preselected ROIs. As compared with other model based methods such as the dynamic causal modeling (DCM) [26], [27], the exploratory nature of the PPI analysis is suitable in the current stage, because the modulation of functional connectivity is still largely unknown.

In the present study, we focused on the interaction effect of two main regions within the well-studied intrinsic networks [28], and explored regions that exhibited modulatory interactions with the two regions within them. We hypothesize that the modulatory interactions may be present even in the resting-state. The resultant regions may either serve as a modulator of the functional connectivity between the two regions within a network or be modulated by the functional connectivity of the two regions within a network. This study will provide insight into the complex relationships between three (or more) brain regions within or between different brain networks.

## Methods

### Subjects

The fMRI data set was derived from the Beijing_Zang dataset of the 1000 Functional Connectomes Project (http://fcon_1000.projects.nitrc.org/) [8]. The dataset originally contained 198 subjects. 192 subjects remained after the removal of subjects due to large head motion. We used the data from the first 64 subjects (40 female/24 male) as a discovery dataset, and the data from the following 64 subjects (43 female/21 male) as a replication dataset. The two samples were analyzed using the same processing procedure, but the group level statistics were conducted independently to assess the reproducibility of the PPI analysis. The last 64 subjects were not used for the current study. The mean age of the subjects in the discovery sample was 21.1 years (range from 18 to 26 years), and the mean age of the subjects in the replication sample was 21.2 years (range from 18 to 26 years). All the subjects were right-handed.

### Scanning Parameters

The MRI data were acquired using a SIEMENS Trio 3-Tesla scanner from Beijing Normal University. 230 whole brain volumes were acquired in the resting-state for each subject using a TR of 2 s. During the resting-state scan, the subjects were instructed to close their eyes, not to fall asleep, and to avoid thinking about anything in particular. The resolution of the fMRI images was 3.125×3.125×3 mm with 64×64×36 voxels. The T1-weighted sagittal three-dimensional magnetization-prepared rapid gradient echo (MP-RAGE) sequence was acquired using the following parameters: 128 slices, TR = 2530 ms, TE = 3.39 ms, slice thickness = 1.33 mm, flip angle = 7°, inversion time = 1100 ms, FOV = 256×256 mm^2^.

### Functional MRI Data Analysis

#### Preprocessing

The fMRI image preprocessing and PPI analysis were conducted using the SPM8 package (http://www.fil.ion.ucl.ac.uk/spm/) under the MATLAB 7.6 environment (http://www.mathworks.com). For each subject, the first two functional images were discarded, resulting in 228 images for each subject. The functional images were motion-corrected and coregistered to the individual subject’s high resolution anatomical image. Next, the subject’s anatomical images were normalized to the T1 template provided by the SPM package in the Montreal Neurological institute (MNI) space. Then, the normalization parameters were used to normalize all the functional images into the MNI space, and the functional images were resampled into 3×3×3 mm^3^ voxels. Finally, all the functional images were smoothed using a Gaussian kernel with 8 mm full-width at half-maximum (FWHM).

#### Spatial ICA

Spatial independent component analysis (ICA) was conducted to define intrinsic networks and ROIs for PPI analysis using the Group ICA of fMRI Toolbox (GIFT; http://icatb.sourceforge.net/) [29]. Twenty components were extracted. The resulting component maps were visually inspected to identify the commonly used intrinsic networks: the DMN, dorsal attention, left and right executive, salience, auditory, primary visual, extrastriate visual, and motor networks [28].

### PPI Analysis

The PPI analysis was conducted using a voxel-wise statistics using the general linear model (GLM) framework in SPM8. By defining two ROIs, PPI analysis identified regions where the connectivity with one selected ROI was correlated with the increasing or decreasing activity of the second selected ROI. Specifically, for each of our PPI analysis, we defined two ROIs within each of the specific intrinsic network derived from the ICA analysis (see Table 1). Thus, the PPI analysis will find regions whose connectivity with one region is modulated based upon the activity of the other region in the same network. This analysis will shed insight into the dynamic change of connectivity of a given network.

**Table 1:** Components and regions of interests defined by the spatial ICA analysis.

Network	Component	Region	Abbr.	MNI Coordinates		
				x	y	z
Default mode network	18	Posterior cingulate gyrus	PCC	3	−52	26
		Medial prefrontal cortex	MPFC	3	58	6
Left Executive network	2	Left superior frontal gyrus	LSFG	−33	22	52
		Left superior parietal lobule	LSPL	−50	−51	50
Right Executive network	17	Right superior frontal gyrus	RSFG	27	28	52
		Right superior parietal lobule	RSPL	36	−66	48
Salience network	14	Left inferior frontal gyrus	LIFG	−48	19	−5
		Right inferior frontal gyrus	RIFG	48	16	−5
Dorsal attention network	20	Left inferior parietal lobule	LIPL	−45	−42	56
		Right inferior parietal lobule	RIPL	48	−39	55
Auditory network	4	Left superior temporal gyrus	LSTG	−62	−1	9
		Right superior temporal gyrus	RSTG	62	−26	16
Extrastriate network	9	Left middle temporal gyrus	LMTG	−50	−65	10
		Right superior temporal gyrus	RMTG	45	−76	10
Motor network	13	Left precentral gyrus	LPCG	−48	−7	54
		Right precentral gyrus	RPCG	45	−13	54

To define ROIs, the z map of each intrinsic network from the ICA analysis was first thresholded at z >2.3, and the coordinates of peak voxels within the brain structures of interest were obtained (see Table 1). For all the components except auditory and extrastriate visual networks, the coordinates represent the peak voxel of the corresponding cluster. The components that were labeled auditory and extrastriate networks extended far beyond the auditory or extrastriate networks, so we chose the peak voxels within the bilateral superior temporal gyrus to represent the auditory network, and we chose the peak voxels within the bilateral middle temporal gyrus as the extrastriate network. Networks that were comprised of only one main cluster were not included in the PPI analysis, e.g. the primary visual network.

A GLM was constructed for each subject to extract ROI time series for PPI analysis. The GLM contained one pseudo-condition with an onset at the middle of the scan. This dummy regressor was only used so that SPM model estimation procedure was properly conducted; however, this dummy regressor had no relationship with further data analyses. The model also included the first eigenvector of the time series from white matter (WM) and cerebrospinal fluid (CSF) masks and six regressors of rigid-body head motion parameters. An implicit high pass filter of 1/100 Hz was used. The subject-specific WM and CSF masks were derived from their own segmented WM and CSF images, with a threshold of 0.99 to make sure that GM voxels were excluded from the masks. When defining ROIs, the first eigenvector within an 8 mm sphere of the ROI center was extracted after the removal of WM, CSF, head motion and low frequency effects.

The BOLD time series of the two ROIs within a network were first deconvolved with the canonical HRF in the SPM8 using a simple empirical Bayes procedure. Thus, the resulting time course represented an approximation of neural activity [25]. Next, the two neural time series were detrended and point multiplied (scalar product), so that the resulting time series represented the interaction of neural activity between the two ROIs. Then that interaction time series was convolved with the HRF, resulting in an interaction variable at the hemodynamic level. The PPI terms were calculated for ROI pairs from each of the intrinsic networks, and separate PPI models were built for each of the subjects using the GLM framework. The GLM model contained two regressors representing the main effects of the two ROI time series, one regressor representing the PPI effect, two regressors representing WM and CSF signals, and six regressors representing head motion effects. An implicit high pass filter of 1/100 Hz was used.

For each PPI analysis of an intrinsic network, a 2nd-level one sample t-test was conducted to make group-level inference. Simple t contrast of 1 or −1 was defined to reveal positive or negative PPI effects, respectively. The resulting clusters were first height thresholded at p<0.001, and the cluster-level false discovery rate (FDR) was corrected at p<0.05 based on random field theory (i.e. topological FDR) [30]. The group level analyses were conducted for the discovery and replication samples independently, and the similarities and differences between the two samples were examined.

Since the introduction of deconvolution of fMRI time series to calculate the PPI term by Gitelman et al. [25], no empirical studies have been conducted to estimate the impact of deconvolution on the PPI results, especially for the resting-state data. Thus, we calculated the PPI terms using raw fMRI time series for each of the PPI analysis and for each subject. The Pearson’s coefficients of the two PPI terms from deconvolved and raw time series were calculated for each of the networks within the discovery and replication samples to assess the similarities. In addition, voxel-wise PPI analysis was conducted for the DMN ROIs using the PPI terms calculated from the raw fMRI time series. Similar group level analyses using one sample t-test were conducted for both the discovery and replication samples.

## Results

### PPI Analysis

The PPI results for the discovery and replication samples are listed in Table 2 and 3, respectively. The resulting clusters that showed consistent effects across the two samples are highlighted in bold.

**Table 2:** PPI results of the discovery sample for each networks.

Label	BA	cluster p (FDR-cor)	Voxels	Peak T	Peak coordinates		
					x	y	z
DMM							
L. Caudate, Caudate Body		0.013	55	4.39	−21	26	7
**L. Medial Frontal Gyrus**	**6**	**<0.001**	**367**	−**6.04**	−**3**	**47**	**31**
**L. Inferior Parietal Lobule**	**40**	**<0.001**	**123**	−**4.94**	−**45**	−**55**	**49**
R. Inferior Parietal Lobule	40	<0.001	183	−4.73	54	−49	49
**L. Middle Frontal Gyrus**	**10**	**<0.001**	**104**	−**4.73**	−**42**	**44**	**7**
**L. Cingulate Gyrus**	**31**	**0.045**	**34**	−**4.64**	−**6**	−**37**	**49**
**R. Superior Frontal Gyrus**	**10**	**0.004**	**66**	−**4.54**	**36**	**56**	**7**
R. Middle Frontal Gyrus	6	0.04	37	−4.12	36	20	55
L Executive							
R. Superior Frontal Gyrus	6	0.042	45	−4.31	12	44	49
R Executive							
R. Precuneus	7	0.019	48	4.59	21	−73	58
**L. Superior Frontal Gyrus**	**6**	**<0.001**	**514**	−**4.98**	−**18**	**26**	**55**
**L. Angular Gyrus**	**39**	**<0.001**	**169**	−**4.86**	−**48**	−**58**	**40**
Attention							
**L. Inferior Parietal Lobule**	**40**	**0.021**	**48**	**4.77**	−**39**	−**49**	**64**
R. Fusiform Gyrus	37	0.008	69	4.3	48	−55	−11
**R. Superior Parietal Lobule**	**7**	**0.04**	**36**	**4.26**	**21**	−**61**	**70**
Salience							
n.s.							
Auditory							
n.s.							
Extrastriate							
R. Parahippocampal Gyrus	30	0.04	37	4.63	33	−55	4
L. Parahippocampal Gyrus	19	0.03	45	4.36	−30	−58	1
**L. Cuneus**	**17**	**0.03**	**53**	**4.32**	−**12**	−**100**	**13**
Motor							
L. Cingulate Gyrus	24	0.001	109	5.1	−6	14	28
L. Precentral Gyrus	43	<0.001	130	4.98	−54	−4	10
R. Middle Frontal Gyrus	9	0.002	79	4.96	33	53	31
R. Inferior Frontal Gyrus	9	<0.001	226	4.91	63	8	19
L. Insula	13	0.001	89	4.75	−33	14	13
**L. Precuneus**	**7**	**0.02**	**47**	**4.36**	−**3**	−**55**	**67**

The clusters of negative modulation are shown as negative peak t values. The clusters are thresholded at p<0.001, and cluster-level false discovery rate corrected at p<0.05. BA, Brodmann’s area; R, right; L, left; n.s., non-significant. The coordinates represents the coordinates in MNI spaces.

**Table 3:** PPI results of the replication sample for each networks.

Label	BA	cluster p (FDR-cor)	Voxels	Peak T	Peak coordinates		
					x	y	z
DMN							
**R. Middle Frontal Gyrus**	**11**	**0.013**	**52**	−**5.14**	**33**	**41**	−**2**
**L. Cingulate Gyrus**	**31**	**<0.001**	**188**	−**5.04**	**−9**	**−25**	**37**
**L. Medial Frontal Gyrus**	**9**	**<0.001**	**144**	**−5.03**	**−6**	**53**	**25**
R. Sub−Gyral	37	0.017	43	−4.95	57	−49	−8
R. Parahippocampal Gyrus	36	0.032	34	−4.66	30	−40	−8
**L. Middle Frontal Gyrus**	**9**	**0.005**	**76**	**−4.55**	**−42**	**20**	**22**
**L. Cingulate Gyrus**	**23**	**0.014**	**49**	**−4.45**	**−3**	**−10**	**31**
L. Anterior Cingulate	33	0.026	37	−4.44	−3	17	22
**L. Inferior Parietal Lobule**	**40**	**0.008**	**59**	**−4.42**	**−45**	**−52**	**61**
L. Middle Temporal Gyrus	22	0.005	71	−4.42	−57	−37	1
**R. Middle Frontal Gyrus**	**46**	**0.014**	**47**	**−4.34**	**45**	**32**	**19**
**L. Inferior Frontal Gyrus**	**47**	**0.04**	**31**	**−4.21**	**−45**	**29**	**−8**
L. Middle Temporal Gyrus	39	0.014	46	−4.19	−48	−73	34
L. Parahippocampal Gyrus	36	0.008	60	−4.15	−30	−40	−11
L Executive							
n.s.							
R Executive							
**L. Superior Frontal Gyrus**	**6**	**0.005**	**81**	**−5.91**	**−18**	**32**	**58**
**R. Superior Frontal Gyrus**	**6**	**0.005**	**75**	**−4.64**	**15**	**44**	**52**
**L. Medial Frontal Gyrus**	**9**	**0.005**	**76**	**−4.52**	**−9**	**47**	**13**
**L. Middle Temporal Gyrus**	**39**	**0.028**	**48**	**−4.13**	**−48**	**−76**	**34**
Attention							
**R. Superior Parietal Lobule**	**7**	**<0.001**	**556**	**5.13**	**21**	**−61**	**70**
R. Middle Frontal Gyrus	6	0.014	63	4.55	30	−4	61
L. Middle Frontal Gyrus	46	0.004	93	−4.89	−45	50	10
R. Superior Frontal Gyrus	9	0.009	70	−4.47	30	53	22
R. Inferior Parietal Lobule	40	0.04	45	−4.36	60	−49	46
Salience							
n.s.							
Auditory							
L. Precentral Gyrus	6	<0.001	242	6.08	−54	−1	22
R. Precentral Gyrus	6	<0.001	148	4.64	63	−1	22
Extrastriate							
**L. Cuneus**	**18**	**0.001**	**98**	**4.87**	**−3**	**−79**	**22**
Motor							
R. Cuneus	18	0.041	43	4.5	18	−82	25
R. Lingual Gyrus	18	<0.001	123	4.43	30	−82	−2
**L. Precuneus**	**7**	**0.043**	**39**	**4.32**	**−9**	**−58**	**70**
L. Cerebellum, Posterior Lobe		0.038	49	3.86	−18	−64	−17
R. Caudate, Caudate Body		0.035	50	−5.06	15	5	19
L. Posterior Cingulate	23	0.035	51	−4.92	−6	−25	22

The clusters of negative modulation are shown as negative peak t values. The clusters are thresholded at p<0.001, and cluster-level false discovery rate corrected at p<0.05. BA, Brodmann’s area; R, right; L, left; n.s., non-significant. The coordinates represents the coordinates in MNI spaces.

**Figure 1:**
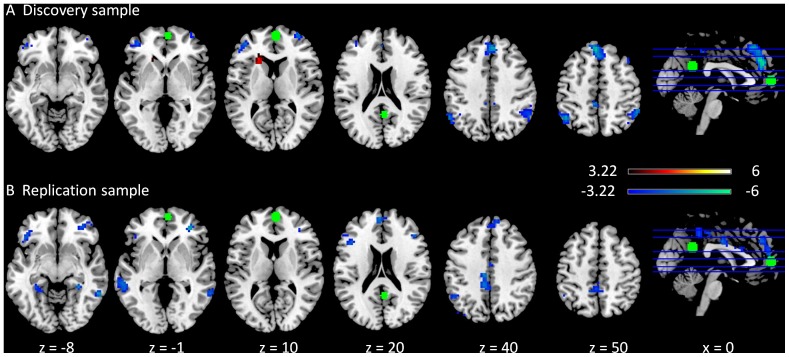
Clusters correlated with positive (in hot) and negative (in cold) modulation effect of the functional connectivity between the two DMN ROIs in the discovery (A) and replication (B) samples. The green circles represent the ROIs used in this PPI analysis. The maps were thresholded at p<0.05 FDR corrected with a heigth threshold of p<0.001. The x and z values represent the coordinates in the MNI space.

A majority of clusters exhibited negative modulatory interactions with the medial prefrontal cortex (MPFC) and the posterior cingulate gyrus (PCC) regions (green circles) of the DMN in both the discovery (Figure 1A) and replication (Figure 1B) samples. Clusters that showed consistent negative modulatory interactions for the two samples were located in the medial frontal gyrus (BA 6), the left inferior parietal lobule (BA 40), the cingulate cortex (BA 23/31), and the anterior portion of the bilateral middle frontal gyrus (BA 9/10/46). The right interior parietal lobule [40], and the right middle frontal gyrus (BA6) clusters only showed negative modulatory interactions in the discovery sample. And the bilateral temporal lobule (BA 22/37/39), the bilateral parahippocampal gyrus (BA 36), and the anterior cingulate gyrus (BA 33) only showed negative modulatory interactions in the replication sample. In contrast, there was also one cluster that revealed a positive modulatory interaction with the MPFC and PCC regions in the discovery sample, which was located in the left caudate body. Although this cluster was not observed in the replication sample when using a cluster-level FDR correction at p<0.05, we observed several small clusters in the caudate when we did not apply a cluster extent threshold.

**Figure 2:**
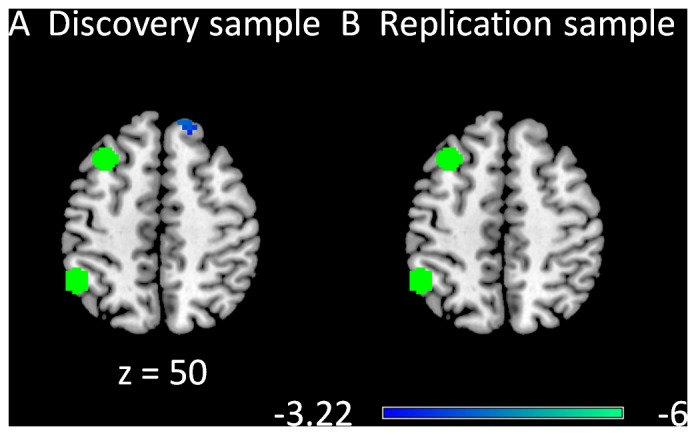
Clusters correlated with positive (in hot) and negative (in cold) modulation effect of the functional connectivity between the two left executive network ROIs in the discovery (A) and replication (B) samples. The green circles represent the ROIs used in this PPI analysis. The maps were thresholded at p<0.05 FDR corrected with a heigth threshold of p<0.001. The z values represent the coordinates in the MNI space.

Only negative modulatory interactions were observed for the left superior frontal gyrus (LSFG) and the left superior parietal lobule (LSPL) regions of the left executive network (Figure 2). In the discovery sample, one cluster located in the right superior frontal gyrus (BA 6) revealed a negative modulatory interaction with the LSFG and LSPL seeds (Figure 2A). No clusters were observed in the replication sample when using a cluster-level FDR correction at p<0.05 (Figure 2B). However, we observed a small cluster within the superior frontal gyrus when a cluster extent threshold was not applied.

**Figure 3:**
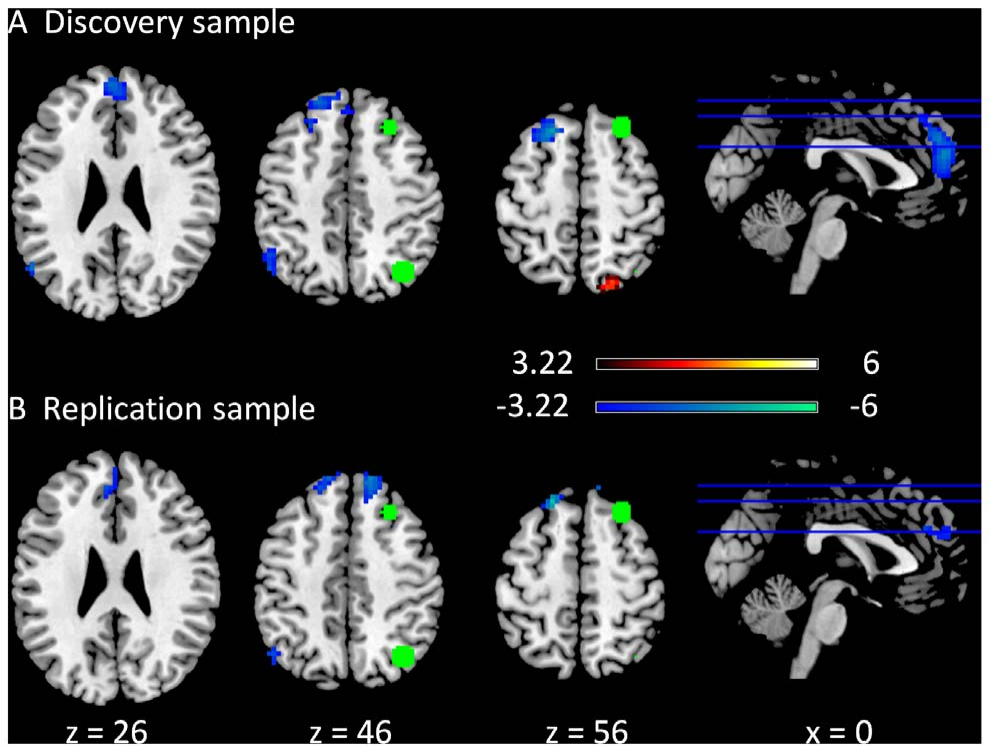
Clusters correlated with positive (in hot) and negative (in cold) modulation effect of the functional connectivity between the two right executive network ROIs in the discovery (A) and replication (B) samples. The green circles represent the ROIs used in this PPI analysis. The maps were thresholded at p<0.05 FDR corrected with a heigth threshold of p<0.001. The x and z values represent the coordinates in the MNI space.

The clusters that revealed negative modulatory interactions with the right superior frontal gyrus (RSFG) and the right superior parietal lobule (RSPL) regions of the right executive network were generally similar between the discovery (Figure 3A) and replication (Figure 3B) samples, including regions from the superior frontal gyrus to the medial frontal gyrus (BA 6/9), and a cluster in the temporal/parietal region (BA 39). In the discovery sample, there was also a cluster that revealed a positive modulatory interaction with the RSFG and RSPL regions, which was localized in the precuneus (BA 7). Similar clusters were not observed in the replication sample even when the cluster correction was not applied.

No suprathreshold clusters showed modulatory interaction effects with the left inferior frontal gyrus (LIFG) and the right inferior frontal gyrus (RIFG) regions of the salience network in both samples.

**Figure 4:**
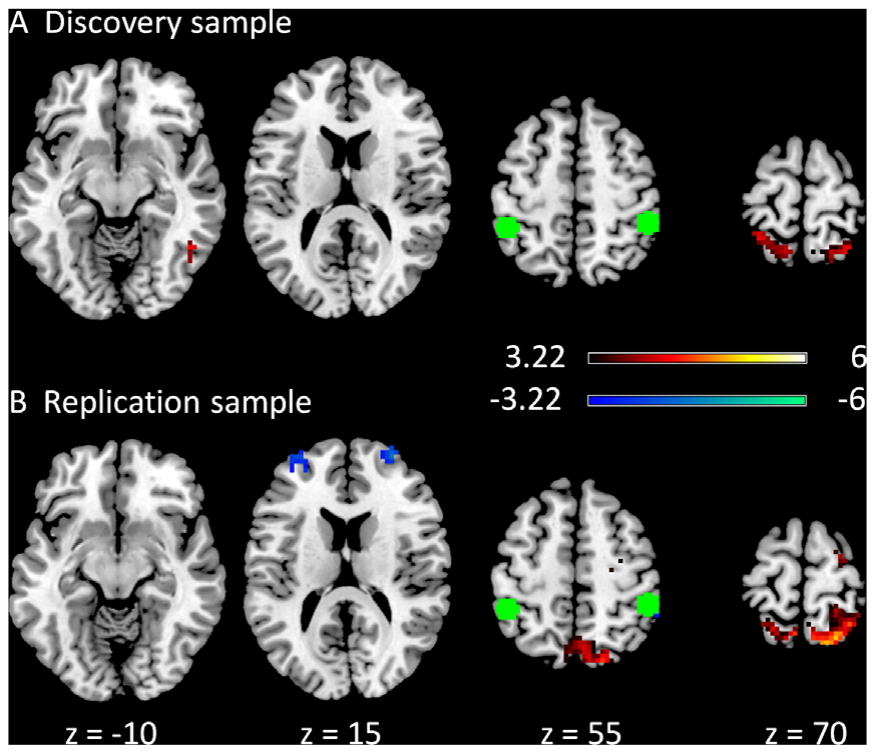
Clusters correlated with positive (in hot) and negative (in cold) modulation effect of the functional connectivity between the two dorsal attention network ROIs in the discovery (A) and replication (B) samples. The green circles represent the ROIs used in this PPI analysis. The maps were thresholded at p<0.05 FDR corrected with a heigth threshold of p<0.001. The z values represent the coordinates in the MNI space.

The clusters that showed modulatory interactions with the left inferior parietal lobule (LIPL) and the right inferior parietal lobule (RIPL) regions of the dorsal attention network in the discovery (A) and replication (B) samples are illustrated in Figure 4. Common clusters that showed positive modulatory interactions in both the samples were located in the superior potion of the parietal lobule (BA 7/40). In the discovery sample, a cluster that was located in the right fusiform gyrus (BA 37) also revealed a positive modulatory interaction with the LIPL and RIPL regions. This cluster was also observed in the replication sample when the cluster level correction was not applied. In the replication sample, a cluster located in the frontal eye field (BA6) also demonstrated a positive modularoty interaction with the LIPL and RIPL regions. In addition, in the replication sample, three clusters also showed negative modulatory interactions with the LIPL and RIPL regions, which were located in the frontal pole regions (BA 9/46), and the inferior parietal lobule (BA 40).

**Figure 5:**
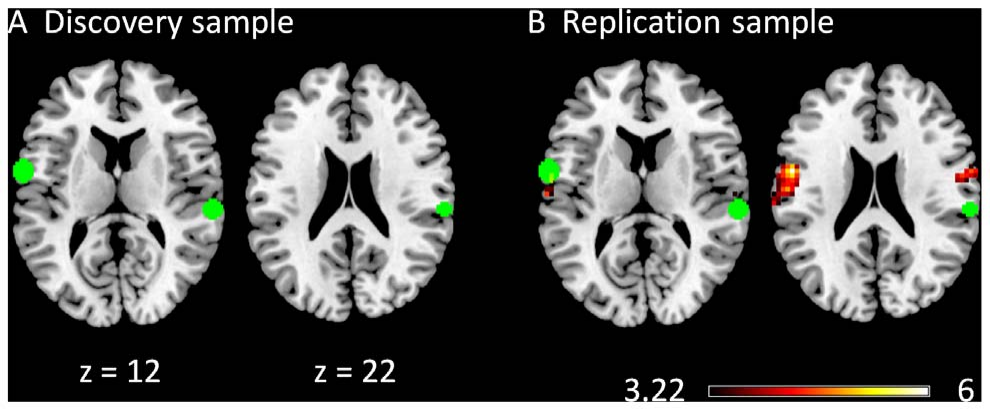
Clusters correlated with positive (in hot) and negative (in cold) modulation effect of the functional connectivity between the two auditory network ROIs in the discovery (A) and replication (B) samples. The green circles represent the ROIs used in this PPI analysis. The maps were thresholded at p<0.05 FDR corrected with a heigth threshold of p<0.001. The z values represent the coordinates in the MNI space.

No clusters revealed positive or negative modulatory interactions with the left superior temporal gyrus (LSTG) and the right superior temporal gyrus (RSTG) regions of the auditory network in the discovery sample (Figure 5A). While in the replication sample, two clusters located in the bilateral precentral gyrus (BA 6) revealed positive modulatory interactions with the LSTG and RSTG regions. These two clusters were also observed in the discovery sample when we did not apply a cluster level threshold.

**Figure 6:**
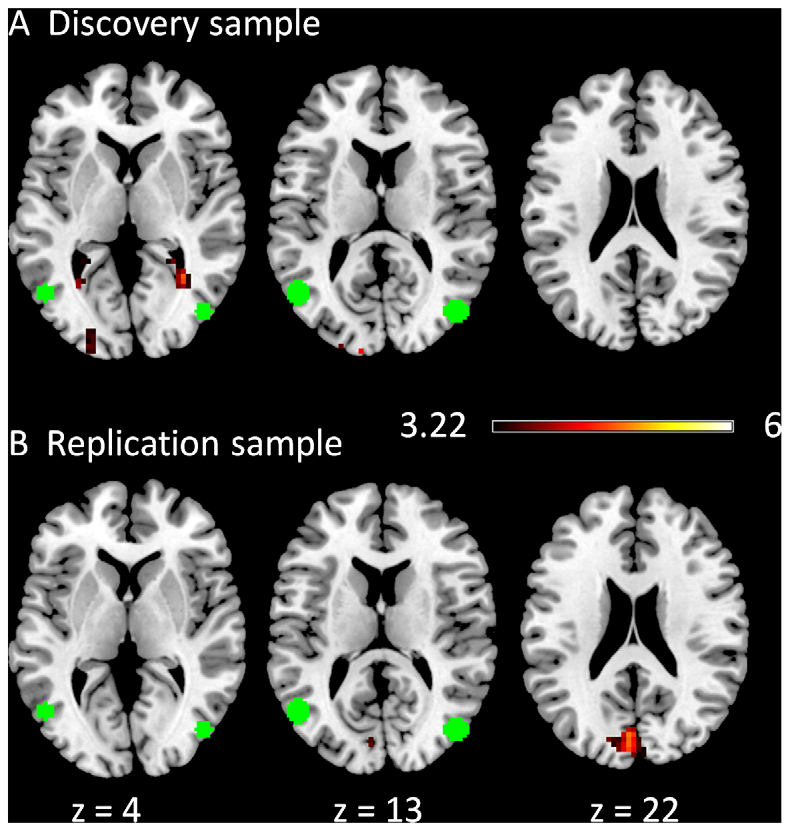
Clusters correlated with positive (in hot) and negative (in cold) modulation effect of the functional connectivity between the two extrastriate network ROIs in the discovery (A) and replication (B) samples. The green circles represent the ROIs used in this PPI analysis. The maps were thresholded at p<0.05 FDR corrected with a heigth threshold of p<0.001. The z values represent the coordinates in the MNI space.

Regions in the cuneus (BA 17/18) revealed consistent positive modulatory interactions with the left middle temporal gyrus (LMTG) and the right middle temporal gyrus (RMTG) regions of extrastriate network in both the discovery (Figure 6A) and the replication (Figure 6B) samples. In addition, two clusters located in the bilateral parahippocampal gyrus (BA 19/30) also revealed positive modulatory interactions with the LMTG and RMTG seeds in the discovery sample. No clusters revealed negative modulatory interactions with the LMTG and RMTG regions.

**Figure 7:**
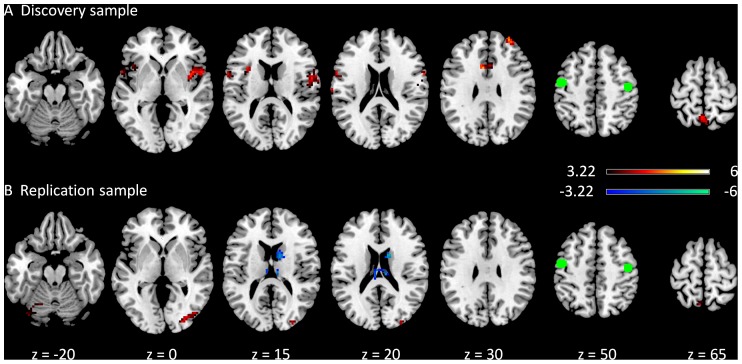
Clusters correlated with positive (in hot) and negative (in cold) modulation effect of the functional connectivity between the two motor network ROIs in the discovery (A) and replication (B) samples. The green circles represent the ROIs used in this PPI analysis. The maps were thresholded at p<0.05 FDR corrected with a heigth threshold of p<0.001. The z values represent the coordinates in the MNI space.

The modulatory interactions with the left precentral gyrus (LPCG) and the right precentral gyrus (RPCG) regions of the motor network revealed different spatial patterns in the discovery (Figure 7A) and the replication (Figure 7B) samples. In the discovery sample, positive modulatory interactions were observed in the bilateral precentral gyrus/insula (BA 9/13/43), the cigulate gyrus (BA 24), the precuneus (BA 7) and the right middle frontal gyrus (BA 9). In the replication sample, positive modulatory interactions were observed in the occipital regions (BA 18), the posterior cerebellum, and the precuneus (BA 7). Only the precuneus region was found in both samples. In addition, the right caudate body and the posterior cingulated gyrus that extended to the thalamus exhibited negative modulatory interaction with the LPCG and RPCG regions in the replication sample.

### The Effects of Deconvolution

**Figure 8:**
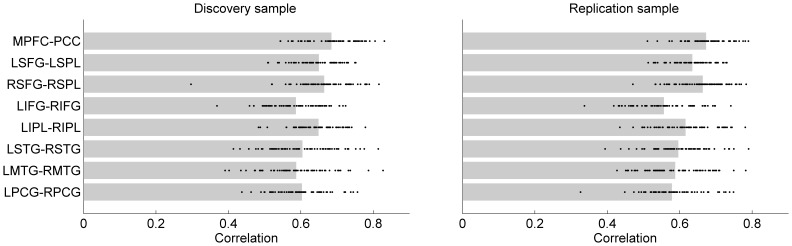
Correlation of the PPI terms calculated using deconvolved versus nondeconvolved time series for each PPI analysis and sample. The bars represent mean Pearson’s correlation across subjects, and each dot represent a single subject.

The correlations of the PPI terms using the deconvolved time series and the raw BOLD time series are demonstrated in Figure 8. In the discovery sample, the mean correlations ranged from 0.59 to 0.68 for the eight networks, and in the replication sample, the mean correlations ranged from 0.56 to 0.67. The correlations were fairly consistent across the eight networks (range within 0.1), however, the variance of mean correlations across networks showed a strong association between the two samples (*r = 0.95, p<0.001*).

**Figure 9:**
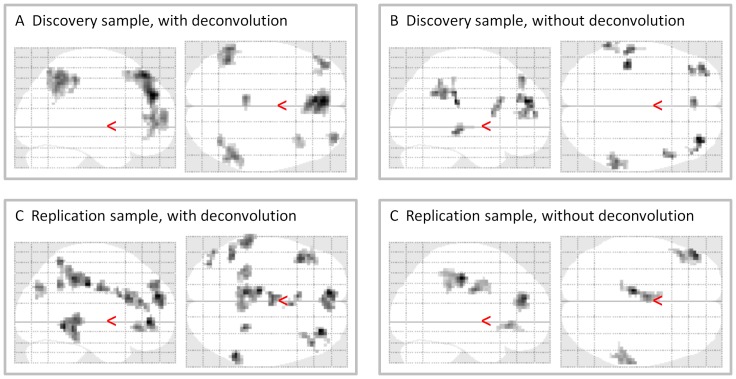
Effects of PPI term calculation on voxel-wise PPI results in the discovery (A, B) and replication (C, D) samples. Effects shown were negative PPI effects of the two DMN ROIs rendered on a glass brain. The maps were thresholded at p<0.05 FDR corrected with a heigth threshold of p<0.001.

Figure 9 demonstrates negative modulatory interactions of the MPFC and PCC regions of the DMN calculated by both the deconvolved and the raw BOLD time series in both the discovery and replication samples, respectively. A glass brain was used to show the spatial distribution of the clusters. In general, the PPI results based on raw BOLD time series showed similar spatial patterns with smaller number of clusters and smaller clusters sizes (Figure 9B and 9D) when compared with the PPI results based on deconvloved time series (Figure 9A and 9C) in both the discovery and replication samples.

**Figure 10:**
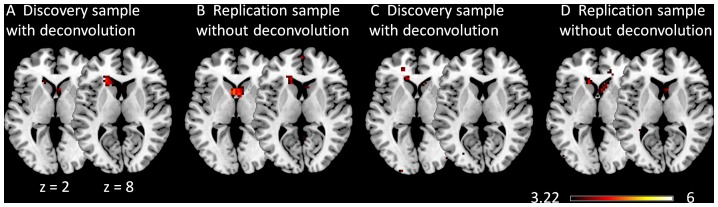
Effects of PPI term calculation on voxel-wise PPI results in the discovery (A, B) and replication (C, D) samples. Effects shown were positive PPI effects of the two DMN ROIs. The maps were thresholded at a heigth threshold of p<0.001 without correction of cluster size. The z values represent the coordinates in the MNI space.

Figure 10 illustrates the positive modulatory interactions of the MPFC and PPC regions of the DMN calculated from both deconvolved and raw BOLD time series using a height threshold of p<0.001 without applying a cluster extent threshold. Small clusters within the caudate regions revealed positive modulatory effects with the DMN regions for the both PPI calculation methods in both samples. However, when a cluster level FDR correction threshold was applied, only the analyses for the discovery sample showed significant modulatory effects in the caudate regions (Figure 10A/10B).

## Discussion

By applying physiophysiological interaction analysis to the resting-state fMRI data, the current study identified regions that were associated with the modulatory interactions of the two regions that represented different brain networks. Seven out of the eight networks showed significant PPI effects in the discovery and/or replication samples. We observed that the ROI pairs in some of the networks such as the extrastriate network, the dorsal attention network, auditory network, and the motor network showed positive modulatory interactions with regions that were functionally related to those network s. In addition, regions in the competing networks, i.e., the DMN and executive network, demonstrated negative modulatory interactions.

### Explanation of PPI Effects

In addition to a simple correlation with one ROI, the PPI effect suggests a non-additive effect among the two ROIs and the resulting cluster. The connectivity from region A to C, and the connectivity from B to C do not satisfy the superposition principle, and implies a nonlinear relationship among those three regions. The nonlinear dynamics of brain connectivity are evident in the resting-state when using electroencephalography (EEG) [31], [32], and are shown to be critical in the emergence of low-frequency fluctuations of the fMRI signals using simulation data [23], [33], [34], [35]. This nonlinear modulatory effect can be explained at the neuronal level by the mechanism of short-term synaptic plasticity (STP), which results in dynamic alterations of synaptic strengths on time scales of milliseconds to minutes [36]. Additionally, it can also explained at the system level where the modulatory effect may serve as a control mechanism from one brain regions to modulate information transmissions between two other regions, which may support attentional gating or switching processes [37], [38].

Given that the analysis uses a correlation approach, the lack of direction in modulation effects is a shortcoming of the PPI results. Therefore, the modulation effect may be due to either of the ROI regions that modulate the connectivity of the other ROI to the resultant region or vice versa. Such information can only be obtained by the existing literatures on neuroanatomy and effective connectivity analysis. Some sophisticated models such as DCM [26], [27] and Granger causality analysis [39] has been shown to provide directional information regarding the modulation effects. These methods may be helpful in the future to study directed modulatory effects.

### PPI Effects of Specific Networks

Positive modulatory interactions were observed in the analyses of the extrastriate network and dorsal attention network. Specifically, the cuneus region showed positive modulatory interactions with the bilateral MTG of the extrastriate network, and the superior parietal lobule and the fusiform gyrus revealed positive modulatory interactions with the bilateral IPL regions of the dorsal attention network. Prior studies have reported that the middle temporal visual areas have reciprocal connections to other visual areas and dorsal parietal regions [40], [41]. In addition, the connectivity between the primary visual area, middle temporal gyrus and parietal regions has been reported to be modulated by extrinsic tasks such as stimulus motion and attention [26], [42], [43], [44]. However, these studies do not explain the neuronal origin of task modulations. Stephan and colleagues have demonstrated a nonlinear modulation of parietal lobe on the connectivity from V1 to V5 [27]. The present results reveal similar nonlinear modulations among regions in the visual and dorsal attention systems. Taken together, these results may suggest an important role of nonlinear modulation to support attentional gating which selectively processes information from lower to higher visual areas [45]. Interestingly, these modulation effects were present even at resting-state conditions where the subjects closed their eyes and were not involved in a specific task. Such observation may suggest that the nonlinear modulation may be intrinsic.

It is of particular interest to examine the modulation effects on the DMN regions, because these regions involve regions that are “active” during the resting-state [46]. An interesting observation is that the left caudate body showed positive modulatory interactions with the MPFC and PCC of the DMN. The caudate has been shown to receive intensive afferent projection from the cortex [47], [48] and modulates the cortical activity through GABAergic (gamma aminobutyric acid) neurons [49]. In the human brain, widely distributed cortical regions have been shown to connect to the caudate via structural [50], [51] and functional connectivity [52]. The amplitude of the local low frequency fluctuations within the caudate exhibited association with the connectivity between the caudate to wide spread cortical regions such as the DMN regions [53]. In addition, Granger causality analysis has shown that the DMN nodes, such as the MPFC and PCC, receive information from most of the brain regions which has been coined as the ‘driven hub’ of the brain [54], [55]. Taken together, it is possible that the caudate regulates both the PCC and MPFC and may coordinate synchronous activities between these regions.

Negative modulatory interactions were mainly observed in the analysis of the DMN and executive networks. Interestingly, the clusters showing negative modulatory interactions in the DMN analysis were mainly located within the executive network, including the bilateral inferior parietal lobule, the bilateral middle/superior frontal gyrus, and the dorsal portion of the medial frontal gyrus (Figure 1). Conversely, the clusters showing negative PPI in left or right executive network analyses were mainly located within the DMN, such as the superior frontal gyrus, the anterior portion of the medial frontal gyrus, and the angular gyrus/middle temporal gyrus (Figure 2 and 3). Given the increasing consensus that the activities of the DMN and task positive networks are negatively correlated [56], [57], [58], [59], the current results further reveal that regions in the DMN and executive networks showed negative nonlinear modulations. The negative PPI effects may suggest that the connectivity between the two nodes of a network is negatively modulated by the regions in the competing network. Alternatively, it is also possible that the connectivity between two regions from competing networks is negatively modulated by another region from the other network, suggesting increased anti-correlations. These nonlinear competing relationships are likely to be mediated by inhibitory neurotransmitters such as GABA, given its critical role in forming anti-correlation neural systems [60], [61], [62].

Positive modulatory interactions were also observed in the PPI analyses of the motor network and the auditory network. These results suggest modulatory interactions among the motor areas, the insula/precentral gyrus, the superior temporal gyrus, and the prefrontal regions. However, these results are less consistent in the two samples compare with other networks. We believe that these modulatory interactions may be due to the functional relevance between these regions [63]. For example, modulation effects such as between the prefrontal cortex and motor areas have been shown in motor task execution [64]. However, further studies are needed to confirm these effects.

### Methodological Considerations

One technical consideration when using PPI is whether a deconvolution step should be implemented prior to the calculation of the PPI term. The deconvolution step was first introduced by Gitelman and colleagues [25], based on the rationale that the calculation of PPI terms using deconvolved “neuronal” time series was less likely to be affected by noises. In line with this notion, our result demonstrates that PPI analysis (in DMN) using the raw BOLD time series generally exhibited smaller cluster size and less number of clusters. These findings suggest that the deconvolution step may be necessary in the PPI analysis to minimize noises. In addition, the current analysis showed fairly consistent correlations between the PPI terms calculated from the deconvolved time series and the PPI terms calculated from the raw BOLD time series. These correlations are smaller than the correlation between psychophysiological interactions reported by Gitelman et al [25]. The reason may be that the calculation of physiophysiological interaction requires deconvolution of two BOLD time series, while the calculation of psychophysiological interaction only requires a deconvolution of one BOLD time series. An interesting observation is that the variability of correlations across different networks is fairly consistent between the discovery and replication samples (*r = 0.95)*, implying that the correlations between the PPI terms using deconvolved and raw time series may reflect the level of inherent noises in different networks.

### Future Directions

The present study illustrates that network dynamics can be captured using the resting-state fMRI data. The modulatory interactions may be used to explain the variations in connectivity over time [12], [13], [14], [15], [16], [17], [18]. Given that the resting-state fMRI datasets are increasingly available [8], [65], the method validated in the present study may provide a novel approach to systematically examine network dynamics in the large-scale brain system [66]. In addition, future studies of modulatory interactions may explain the individual [67], and group level differences in functional connectivity as well as yield insight regarding mental diseases [68]. Since the modulation of functional connectivity has been shown to vary across tasks [69], future studies are needed to investigate the similarities and differences in nonlinear dynamics between the resting-state and during specific task conditions. Secondly, since the current study preselected a total of 8 pairs of regions representing the eight brain networks which provides how regions within a network interact with other regions, examining the modulatory interactions of two regions from different networks may also provide valuable information. For example, Chang and Glover have shown that the task positive network such as the supplementary motor area, parietal cortex, and dorsolateral prefrontal cortex have higher variance in connectivity with the default mode regions [12]. How the relationships between the task positive network and DMN are modulated by other regions may provide important clues regarding the competing nature of the two networks [56].
